# *Drosophila* p120-catenin is crucial for endocytosis of the dynamic E-cadherin–Bazooka complex

**DOI:** 10.1242/jcs.177527

**Published:** 2016-02-01

**Authors:** Natalia A. Bulgakova, Nicholas H. Brown

**Affiliations:** The Gurdon Institute andDept of Physiology, Development and Neuroscience, University of Cambridge, Tennis Court Rd, Cambridge CB2 1QN, UK

**Keywords:** E-cadherin trafficking, Epithelial morphogenesis, Cell adhesion

## Abstract

The intracellular functions of classical cadherins are mediated through the direct binding of two catenins: β-catenin and p120-catenin (also known as CTNND1 in vertebrates, and p120ctn in *Drosophila*). Whereas β-catenin is crucial for cadherin function, the role of p120-catenin is less clear and appears to vary between organisms. We show here that p120-catenin has a conserved role in regulating the endocytosis of cadherins, but that its ancestral role might have been to promote endocytosis, followed by the acquisition of a new inhibitory role in vertebrates. In *Drosophila*, p120-catenin facilitates endocytosis of the dynamic E-cadherin–Bazooka subcomplex, which is followed by its recycling. The absence of p120-catenin stabilises this subcomplex at the membrane, reducing the ability of cells to exchange neighbours in embryos and expanding cell–cell contacts in imaginal discs.

## Abstract (precis)

**Summary:** In *Drosophila*, p120-catenin facilitates endocytosis of the dynamic E-cadherin, which is followed by its recycling. Consequently, p120-catenin regulates cell shape and promotes the ability of cells to exchange neighbours.

## INTRODUCTION

Cell–cell adhesion physically links cells within stable cell sheets, while also contributing to dynamic morphogenetic events such as directional proliferation, cell sorting and cell shape regulation (Stepniak et al., 2009). Transmembrane cadherin proteins are major mediators of cell–cell adhesion, which is mediated through binding between extracellular domains of identical cadherins on adjacent cells surfaces (van Roy and Berx, 2008).

We recently discovered that there are two subcomplexes of epithelial cadherin (E-cadherin, also known as Shotgun in *Drosophila*; hereafter E-cad) at cell–cell junctions in the embryonic epidermis of *Drosophila* (Bulgakova et al., 2013). One subcomplex (‘immobile’) does not recover in fluorescence recovery after photobleaching (FRAP) assays, whereas the second (‘mobile’) does. The mobile E-cad subcomplexes contain the scaffolding protein Bazooka (Baz, also known as Par-3). The recovery of the mobile E-cad subcomplex (E-cad–Baz) occurs through mobility within the plasma membrane and a process that requires endocytosis. E-cad–Baz functions to regulate the exchange of neighbours within the epithelium. This raised the question of whether the dynamic behaviour of this E-cad–Baz subcomplex contributes to its function.

Endocytosis of cadherins regulate adhesion strength and remodelling (Classen et al., 2005; Levayer et al., 2011; Troyanovsky et al., 2006). p120-catenin (also known as CTNND1 in vertebrates, and p120ctn in *Drosophila*) regulates cadherin endocytosis (Troyanovsky, 2009) by directly binding the cadherin intracellular domain and masking motifs necessary for endocytosis (Nanes et al., 2012). The p120-catenin family in vertebrates includes three additional proteins: δ-catenin (also known as CTNND2), ARVCF and p0071 (also known as PKP4) (Pieters et al., 2012). All four family members stabilise cadherins at the cell surface (Hatzfeld, 2005). p120-catenin, δ-catenin and ARVCF share some functional overlap, as overexpression of one can rescue loss of another in cultured cells (Davis et al., 2003).

Although p120-catenin is highly conserved in metazoans, *Drosophila* and *C. elegans* lacking their single p120-catenin family member are viable and fertile (Myster et al., 2003; Pettitt et al., 2003). Nonetheless, loss of p120-catenin results in developmental defects in *Drosophila*, including slowed morphogenetic movements during dorsal closure (Fox et al., 2005) and retinal patterning defects (Larson et al., 2008).

Here, we report a new and surprisingly strong phenotype caused by the absence of p120-catenin, namely the severe reduction of E-cad endocytosis, which demonstrates that regulating cadherin endocytosis is a general function of p120-catenin.

## RESULTS AND DISCUSSION

### p120-catenin is required for endocytosis of a mobile E-cad–Baz subcomplex

**Fig. 1.**
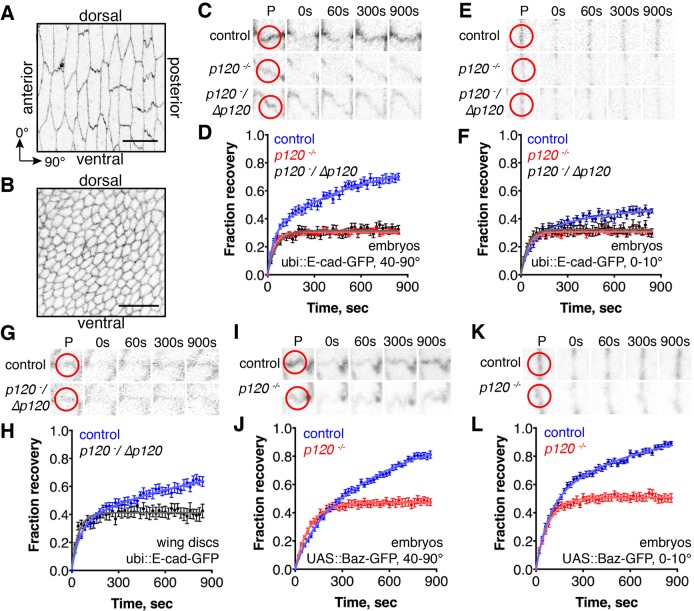
**p120-catenin is required for the slow component of E-cad and Baz recovery in FRAP.** (A,B) ubi::E-cad–GFP distribution in the stage 15 embryo (A) and wing disc (B). The orientation within the embryo and wing (A,B), and direction of 0° and 90° border angles (A) are indicated. Scale bars: 10 µm. (C–H) ubi::E-cad–GFP FRAP at 40–90° (C,D) and 0–10° borders (E,F) in the embryonic epidermis, and in wing discs (G,H). (I–L) UAS::Baz–GFP driven by engrailed::Gal4 FRAP at 40–90° (I,J) and 0-10° borders (K,L) in the embryonic epidermis. Examples of recovery are shown in C,E,G,I and K. Red circles on the prebleached frame (P) outline the bleached spots. Average recovery curves (mean±s.e.m., *n*=13–24; for specific *n* values see Table S1) and the best fit curves (solid lines) are shown in D,F,H,J and L.

To examine the role of p120-catenin, we utilised two *Drosophila* epithelia. First, embryonic epidermal cells at a stage when cell divisions and major morphogenetic movements are completed (referred to as ‘stage 15’). These cells are elongated along the dorso-ventral axis of the embryo and have an asymmetric distribution of E-cad, with more E-cad at cell–cell borders perpendicular to the dorso-ventral axis than on those parallel to it (Fig. 1A). Second, we used epithelial cells in wing imaginal discs from third-instar larvae, which are dividing and have a uniform E-cad distribution (Fig. 1B).

We measured the FRAP of ubi::E-cad–GFP in the epithelia of wild-type embryos and embryos homozygous for the *p120-catenin^308^* null allele (ubi denotes the ubiquitously expressed *Ubiquitin-63E* promoter) (Myster et al., 2003). Owing to the asymmetric distribution of E-cad at stage 15, we separated the data for cell–cell borders with 40–90° and 0–10° angles relative to the dorso-ventral axis (referred to as ‘40–90° borders’ and ‘0–10° borders’). Only a fraction of ubi::E-cad–GFP recovered in stage 15 cells, due to the presence of immobile E-cad, and the recovery was best fitted by a biexponential model composed of two processes (Fig. 1C–F; Table S1). Previously, we have demonstrated that the fast process is diffusion, as its half-time depends on the size of bleached spot, and the slower process occurs through endocytosis, as it is abolished by expression of dominant-negative dynamin and does not depend on the size of bleached spot (Bulgakova et al., 2013; Sprague and McNally, 2005). In wing discs, the recovery of ubi::E-cad–GFP behaved similarly, with an immobile fraction and recovery best fitted by a biexponential model with half-times indistinguishable from stage 15 cells, suggesting that E-cad recovery also occurred by diffusion and an endocytosis-dependent process (Fig. 1G,H; Table S1). The recovery of ubi::E-cad–GFP in animals homozygous for *p120-catenin^308^* (*p120ctn^−^*^/*−*^) or with this allele in *trans* to a deficiency (*p120ctn^−^*/Δ*p120ctn*) showed identical phenotypes, best fitted by single exponential models containing only the rapid diffusive component (Fig. 1C–H; Table S1), indicating that the slow endocytosis-dependent recovery of E-cad is either absent or reduced to a level below what is detectable in these FRAP experiments. Thus, we conclude p120-catenin is required for E-cad recovery through endocytosis in both epithelia.

To discover the fate of the mobile E-cad that no longer exchanged in the absence of p120-catenin, we examined Baz, as previous work has shown that at stage 15 it is only associated with mobile E-cad and not with immobile E-cad (i.e. Baz and E-cad coimmunoprecipitate, Baz has the same dynamics as E-cad in FRAP, but fully recovers; and Baz downregulation reduces mobile E-cad levels without affecting immobile E-cad; Bulgakova et al., 2013). In the control wild-type epithelia, Baz–GFP fully recovered in FRAP assays in a manner best fitted by biexponential models with half-times indistinguishable from E-cad, whereas in *p120-catenin* mutants Baz–GFP was present at adherens junctions and only the fast diffusion half recovered (Fig. 1I–L; Table S1).

**Fig. 2.**
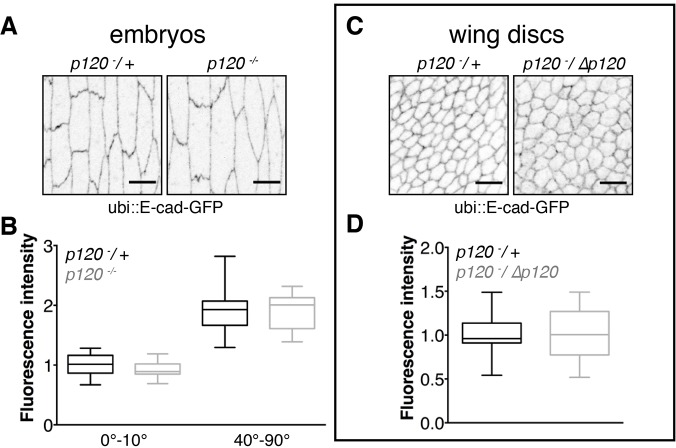
**p120-catenin does not affect the amount of ubi::E-cad–GFP in stage 15 embryos and wing discs.** (A–D) ubi::E-cad–GFP amounts in stage 15 embryos (A,B) and wing discs (C,D). Examples of direct fluorescence of ubi::E-cad–GFP (A,C) are shown. Scale bars: 5 µm. The quantifications are shown in B and D as box plots (*n*=21 in B and 19 in D). The box represents the 25–75th percentiles, and the median is indicated. The whiskers show the minimum and maximum values.

The normal slow E-cad–Baz recovery could occur by replacement of endocytosed proteins with newly synthesised proteins or by recycling. The former case predicts that E-cad should accumulate to higher than normal levels when removal by endocytosis is absent or reduced in the *p120-catenin* mutant. However, contrary to this, the amount of endogenous E-cad was reduced in *p120-catenin* mutant embryos (Fig. S1). This might be explained by p120-catenin regulation of E-cad transcription or mRNA levels (Liu et al., 2014; Stefanatos et al., 2013). To exclude this effect, we expressed E-cad–GFP from another promoter, the ubiquitously expressed *Ubiquitin-63E* (ubi) promoter and found that the amount of E-cad–GFP in *p120-catenin* mutants was indistinguishable from wild type (Fig. 2A–D). Thus, even in the absence of p120-catenin, the insertion of newly synthesised E-cad into the adherens junction is balanced by its removal, and we infer that the normal slow E-cad–Baz recovery occurs by p120-catenin-dependent E-cad endocytosis followed by E-cad recycling.

### Two pathways for E-cad endocytosis

**Fig. 3.**
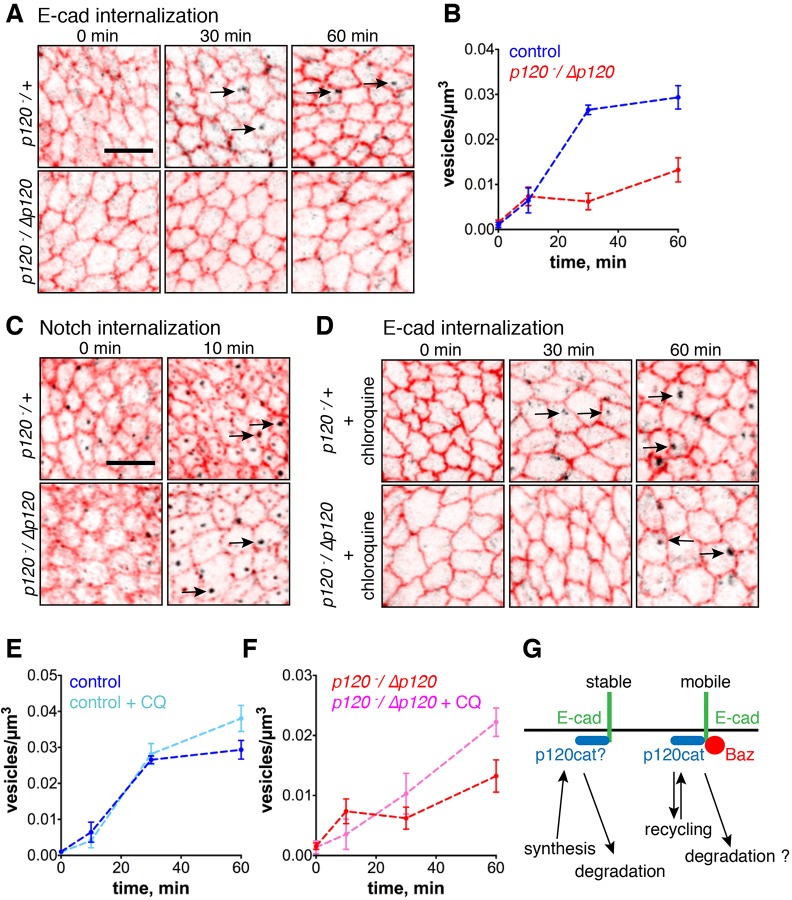
**p120-catenin is not required for internalisation of E-cad for degradation.** (A–F) Pulse-chase labelling of E-cad (A,B), E-cad in presence of chloroquine (CQ) (D–F) and Notch extracellular domains (C) in control and *p120-catenin* mutant wing discs (see Materials and Methods). Examples of immunofluorescence at different time points after pulse-chase labelling are shown in A,C and D, and quantifications of vesicle numbers are in B, E and F (mean±s.e.m., *n*=4 in control without CQ, and *n*=5 for all other datapoints). *z*-stacks were collected every 0.38 µm. Two sets of five images were projected to collect the signal in the 1.9 µm around the adherens junctions (red) and the next 1.9 µm basal to that (black). Arrows indicate examples of intracellular vesicles. Scale bars: 5 µm. (G) The model of two pathways of E-cad endocytosis in *Drosophila* cells. Endocytosis of the E-cad–Baz subcomplex is p120-catenin-dependent and results in E-cad recycling. The second pathway is p120-catenin-independent, results in E-cad degradation and affects either only immobile E-cad or both subcomplexes (uncertainty indicated with ‘?’). It is unclear whether p120-catenin associates with both E-cad subcomplexes or only with E-cad–Baz.

To verify p120-catenin-dependent E-cad endocytosis, we directly measured endocytosis by briefly labelling wing discs with an antibody against the E-cad extracellular domain, and measuring its internalisation at 10, 30 and 60 min after labelling. In the control (*p120^−^*^/*+*^), the number of intracellular vesicles containing anti-E-cad antibody reached 0.026±0.001 vesicles/µm^3^ (mean±s.e.m., *n*=4) by 30 min and was modestly increased at 60 min (Fig. 3A,B; Table S2); this data was best fitted by an exponential model with an 18-min half time, consistent with the FRAP slow-process half time (Tables S1, S2). In *p120-catenin* mutants, the number of vesicles was significantly reduced (*P*<0.0001): very few were detected at 30 min (0.006±0.002 vesicles/µm^3^, mean±s.e.m., *n*=5), and although the number doubled by 60 min, it remained substantially lower than that for the control (Fig. 3A,B). In the mutant, the data was best fitted by a linear model (Table S2), and therefore it was not possible to calculate the half time of the endocytosis rate. However, comparing vesicle number at 30 min and the rate of increase between 0 and 30 min suggests that the endocytic rate is reduced at least fourfold in *p120-catenin* mutants (Table S2). The effect of removing p120-catenin was specific for E-cad, as Notch was internalised normally (Fig. 3C).

Next, we tested whether some E-cadherin was targeted for degradation in the absence of p120-catenin, as suggested by the normal amount of ubi::E-cad–GFP in *p120-catenin* mutants. To be able to visualise this pool, we blocked lysosomal degradation with the lysosomotropic agent chloroquine, which inhibits proteolytic degradation in lysosomes by raising the pH (de Duve et al., 1974). In wild-type epithelia, the number of vesicles was not changed by chloroquine at 30 min, but was increased at 60 min (*P*<0.1, Fig. 3D,E; Table S2). A similar increase in vesicle number at 60 min was observed in *p120-catenin* mutants (*P*<0.1, Fig. 3D,F; Table S2). Given that similar amounts of E-cadherin are slowly targeted for degradation in both wild type and *p120-catenin* mutants, this confirms that our predicted p120-catenin-independent pathway internalises E-cad for degradation. It is not possible to tell whether the few vesicles detected without chloroquine in *p120-catenin* mutants are recycling endosomes or vesicles on the degradation route. If they are the former, then this would suggest some recycling does occur in the absence of p120-catenin. A fourfold reduction of the endocytic rate will appear as no recovery in the FRAP timeframe (Table S1). Therefore, we conclude that the endocytosis of E-cad–Baz for recycling is strongly reduced, perhaps eliminated, in the absence of p120-catenin.

Thus, our experiments reveal two independent pathways for removal of E-cad from the cell surface. The first pathway endocytoses E-cad–Baz for recycling and involves p120-catenin. The second is p120-catenin-independent and targets E-cad for lysosomal degradation (Fig. 3G). The latter pathway might affect immobile E-cad only or both subcomplexes. Evidence suggests that vertebrate cells also have p120-catenin-independent endocytic pathways, for example, a VE-cadherin mutant that cannot bind p120-catenin and lacked the endocytic motif is stabilised at the cell surface, but its surface levels are the same as wild type (Nanes et al., 2012).

In vertebrate cells, p120-catenin family members have a clear role in masking endocytic motifs within the cadherin cytoplasmic tail, yet internalisation of *Drosophila* E-cad was not increased in the absence of p120-catenin. The simplest way to explain this difference is that *Drosophila* E-cad lacks the endocytic motif. Two sequence motifs, LL and DEE, are crucial for internalisation of vertebrate cadherins (Miyashita and Ozawa, 2007; Nanes et al., 2012). The LL motif is absent from *Drosophila* E-cad, and is only found in vertebrate cadherins (Fig. S2). The DEE motif is replaced in *Drosophila* E-cad by a similarly acidic EDE (Fig. S2). Fusion of the core p120-catenin-binding region of *Drosophila* E-cad to IL-2R promotes its internalisation in human endothelial cells (Nanes et al., 2012); this might be mediated by p120-catenin-dependent internalisation or interaction with endocytic machinery unique to vertebrates.

Although we lack evidence for p120-catenin repressing endocytosis in *Drosophila*, there is good evidence that it can promote endocytosis in mammalian cells, by binding Numb and linking it to α-adaptin (Sato et al., 2011). This mechanism is distinct from that in *Drosophila*, as we did not find any changes in E-cad–Baz endocytosis in the absence of Numb (Fig. S3). Taken together, we favour the idea that p120-catenin has an ancestral role of linking cadherins to the endocytic machinery, and additional endocytic motifs arose within the p120-catenin-binding site on cadherins during vertebrate evolution.

### E-cad endocytosis through p120-catenin is required for cell mobility, junctional stability and cell shape regulation

**Fig. 4.**
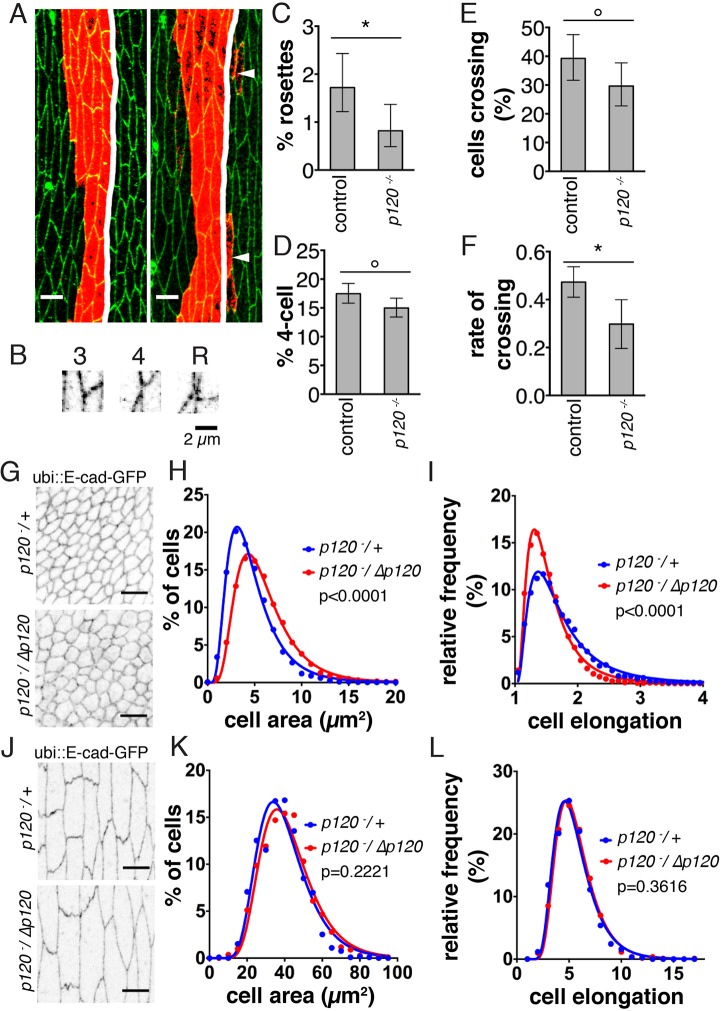
**p120-catenin is required for multicellular rosettes and crossing of segment boundaries by cells in stage 15 embryos and for cell shape in wing discs.** (A) Stripes of *engrailed::Gal4* driving UAS::myr–GFP (red) with cell outlines (anti-E-cad, green) shown. In the example on the left, no cells are outside of the segment boundary (white line). In the example on the right, pairs of cells (arrowheads) are outside of the segment boundary. (B). Examples of three-cell (3), four-cell (4) and five-cell contacts (rosette, R). (C,D) Percentage of rosettes (C) and four-cell contacts (D) between the labelled cells at the boundary and their anterior neighbours. (E) Percentage of stripes with cells expressing the UAS::myr–GFP on the wrong side of the segment boundary. (F) Relative rates of cell pair crossing. In each case the mean±95% confidence interval is shown (*n*=135 segments from 45 embryos). *P<0.01, °*P*<0.05 (Chi-square test in C–E and F-test in F). (G–L) Imaginal discs (G) and stage 15 epidermis (J) from controls (*p120^−^*^/+^) and *p120-catenin* mutants (*p120^−^*/Δp120) with the cell outlines (direct fluorescence of uni::E-cad–GFP) shown. Distributions of apical cell surface areas in wing discs (H) and stage 15 cells (K), and cell elongation (ratio of the length of the long to short axes) in wing discs (I) and stage 15 cells (L) in control (blue) and *p120-catenin* mutants (red). The binned data points (dots) and the best fit lognormal distributions (lines) are shown. In H–I, *n*=10,363 cells from 19 discs in control, and *n*=7985 cells from 19 discs in p120-catenin mutants. In K–L, *n*=1189 cells from 21 embryos in control, and *n*=1116 cells from 21 embryos in p120-catenin mutants. *P* values show the probabilities of the distributions being the same.

We next examined the consequences of E-cad–Baz becoming stabilised at the cell surface. We used ubi::E-cad–GFP to focus on phenotypes caused by the role of p120-catenin in E-cad endocytosis, rather than E-cad expression. Previously, we have reported that reduction of E-cad–Baz levels lowers junction stability, as more contacts form between five or more cells (rosettes) and more cells aberrantly cross segment boundaries (Bulgakova et al., 2013). We examined the lateral cells in the posterior half of each segment. Rosettes between labelled cells at the segment boundary and their anterior neighbours were decreased by 52% in *p120-catenin* mutants (*P*=0.0074, Fig. 4A–C), and accompanied by reduced four-cell contacts (14% reduction, *P*=0.0239, Fig. 4A,B,D). Similarly, the proportion of cell pairs that had crossed the segment boundary, and the relative rate of crossing, were reduced by 25% and 37%, respectively (*P*=0.0480 and *P*=0.0079, Fig. 4A,E,F). As loss of p120-catenin produced an opposite effect to reduction of E-cad–Baz levels, we conclude that it has increased the function of E-cad–Baz.

The stablisation of cells within epithelia could explain other p120-catenin mutant phenotypes, such as defects in retinal patterning and rates of cell movement during dorsal closure (Fox et al., 2005; Larson et al., 2008). Combined loss of p120-catenin-binding sites and endocytic motifs from vertebrate cadherins reduced cell migration upon VEGF induction (Nanes et al., 2012) and disrupted morphogenetic movements in *Xenopus* embryos (Ciesiolka et al., 2004), suggesting that the function of p120-catenin in cell rearrangements is conserved between *Drosophila* and vertebrates.

Next, we tested whether the absence of p120-catenin caused defects in wing discs, measuring apical cell surface area, elongation (the ratio of the lengths of the long and short axes of the best-fit ellipse) and the neighbouring cell number. Apical cell surface area and elongation were best fitted by lognormal distributions. Loss of p120-catenin increased apical cell surface area, changing the centre of the distribution from 3.13±0.04 µm^2^ to 4.31±0.03 µm^2^ (best-fit median±standard error of median estimate, *n*=10,363 cells from 19 discs in control, and *n*=7985 cells from 19 discs in p120-catenin mutants, *P*<0.0001, Fig. 4G,H), and caused the cells to round up, changing the centre of the distribution from 1.37±0.007 to 1.31±0.005 (best-fit median±standard error of median estimate, *n*=10,363 cells from 19 discs in control, and *n*=7985 cells from 19 discs in p120-catenin mutants *P*<0.0001, Fig. 4G,I). The numbers of cell neighbours did not change (Fig. S4A), suggesting that overall tissue packing was unaffected. Similar changes did not occur in the embryonic epidermis (Fig. 4J–L; Fig. S4B). The increased apical area and rounding up following stabilisation of surface E-cad–Baz could arise from increasing the fraction of E-cad engaged in adhesion or increasing its association with the cortical actin cytoskeleton.

To summarise, we have demonstrated that at least one function of p120-catenin is similar between vertebrates and *Drosophila* – that p120-catenin promotes endocytosis of E-cad. Furthermore, we have found that E-cad endocytosis induced by p120-catenin is followed by E-cad recycling, and is required for cell shape regulation and cell rearrangements during morphogenesis.

## MATERIALS AND METHODS

### Fly stocks

*p120ctn^308^*, *Df(2R)M41A8*/ SM1 (Δ*p120*/ SM1), *numb^1^*/CyO, Df(2L)γ7/ CyO (Δ*numb*/ CyO), en::Gal4, UAS::myr-GFP and *CycA^C8LR1^*/TM3 (Bloomington stock numbers 6664, 740, 4096, 6368, 30,564, 32,197, 6627, respectively), ubi::E-cad-GFP (Kyoto stock number 109,007) and UAS::Baz-GFP (Krahn et al., 2010) were used. The *p120-catenin* mutants examined lacked both maternal and zygotic contributions.

### FRAP

Imaging was performed on lateral epidermal cells (segments A2–A4) at late stage 15, following dorsal closure completion, and in the posterior-dorsal quadrant of wing pouches from third-instar larval imaginal discs. Live imaging was performed in embryos as described in Bulgakova et al., 2013, and in wing discs as described in Aldaz et al., 2010. FRAP was performed and analysed as described in Bulgakova et al., 2013.

### Fluorescence intensity quantification, and cell shape and neighbour analysis

Rat anti-E-cad (1:100, DCAD2, DSHB) and Cy3-conjugated anti-rat-IgG (Invitrogen) were used. All imaging was performed with an Olympus FV1000 upright confocal microscope using 100×/1.40 Oil UPlanSApo objective. A total of 21 embryos and 19 wing discs of each genotype were used. Quantifications were performed using Packing Analyzer v2.0 (Aigouy et al., 2010) and custom MATLAB scripts (http://uk.mathworks.com/), which are available upon request. The values for each type of border in a single animal were averaged and used as individual data points to compare datasets with the non-parametric Mann–Whitney test. The binned distributions of apical cell areas and elongation were fitted with lognormal distributions, and cell neighbour numbers with normal distributions using GraphPad Prism software (http://www.graphpad.com/). An F-test was used to compare datasets.

### Pulse-chase assay

Discs were dissected in M3 medium (Sigma), incubated with anti-E-cad (1:200, DCAD2, DSHB) or anti-Notch (1:50, C458.2H, DSHB) antibodies in M3 medium at 4°C for 1 h. Then, the antibody was washed out with cold M3 medium, and discs were incubated in fresh M3 medium with or without 200 µM chloroquine (C-6628, Sigma). Five discs per time point (four discs in control without chloroquine) were fixed in 4% formaldehyde in PBS after labelling and stained with Alexa-Fluor-488-conjugated secondary antibodies (Jackson ImmunoResearch Laboratories). Quantification of vesicle numbers was performed with the 3D ObjectCounter Plugin in Fiji.

### Segment border crossing and multicellular rosette quantification

The number of cells outside of the segment boundaries and types of cell–cell contacts formed by the cells at segment boundaries was analysed as described in Bulgakova et al., 2013.
